# Editorial for the Special Issue “Materials under High Pressure”

**DOI:** 10.3390/ma17010017

**Published:** 2023-12-20

**Authors:** Chuanting Wang, Yuanfeng Zheng, Xiaoguang Qiao, Wenhui Tang, Shuhai Zhang, Yong He

**Affiliations:** 1 School of Mechanical Engineering, Nanjing University of Science & Technology, Nanjing 210094, China; ctwang@njust.edu.cn; 2 State Key Laboratory of Explosion Science and Technology, Beijing Institute of Technology, Beijing 100081, China; zhengyf@bit.edu.cn; 3 School of Materials Science and Engineering, Harbin Institute of Technology, Harbin 150001, China; xgqiao@hit.edu.cn; 4 College of Science, National University of Defense Technology, Changsha 410073, China; wenhuitang@163.com; 5 School of Environment and Safety Engineering, North University of China, Taiyuan 030051, China; zsh93y@nuc.edu.cn

## 1. Introduction

The high-pressure-related problems of materials constitute a field at the confluence of several scientific disciplines. High pressure can be generated by die compression, high-velocity impact, or explosions. The understanding of materials under extreme high pressure, including flow, plastic deformation, phase transformation, fracture, temperature rise, and chemical reactions. 

This Special Issue on the “Materials under High Pressure” collected recent research findings on the high-pressure-related problems of various materials. A collection of fourteen peer-reviewed research articles was included in this Special Issue. The main topics covered include processing technology, characterization, testing, theoretic modeling, and simulation.

## 2. Dynamic Mechanical Properties and Constitutive Model of Materials

Wang et al. [1] investigated the microstructures and deformation mechanism of hetero-structured pure Ti under various high strain rates. The results indicated that as the strain rate increased, the dominance of the dislocation slip decreased, while deformation twinning became more prominent. In addition, nanoscale twin lamellae were activated within the grain with a size of 500 nm at a strain rate of 2000 s^−1^. The modified Hall–Petch model was obtained, with the obtained value of *K*_*twin*_ serving as an effective metric for this relationship. Zhang et al. [2] prepared Zr/PTFE and Ti/PTFE composites via cold isostatic pressing and vacuum sintering. They investigated the static and dynamic compressive mechanical properties of these composites at various strain rates. The results showed that the introduction of zirconium powder and titanium powder could increase the strength of the material under dynamic loading. Meanwhile, a modified Johnson–Cook (J–C) model considering strain and strain rate coupling was proposed.

## 3. Dispersion Characteristics of Materials Driven by Explosion

Materials would exhibit various dispersion characteristics when driven by explosion. Sun et al. [3] presented an investigation on the dispersal characteristics of the cylindrically packed material of dry powder particles driven by explosive load. By establishing a controllable experimental system under laboratory conditions and combining with near-field simulation, the particle dispersal process was described. The characteristic parameters of radially propagated particles were explored under different mass ratios of particle to charge (M/C). Results indicate that when the charge mass remains constant, an increase in M/C leads to a decrease in dispersed jet number, void radius, and maximum velocity, wherein the maximum velocity correlates with calculations by the porous Gurney model. The case of the smaller M/C always has a higher outer-boundary radius and area expansion factor. 

He et al. [4] investigated the axial distribution of the initial velocity and direction angle of double-layer prefabricated fragments after an explosion. A three-stage detonation driving model of double-layer prefabricated fragments was proposed. It was shown that the energy utilization rate of detonation products acting on the inner-layer and outer-layer fragments were 69% and 56%, respectively. The deceleration effect of sparse waves on the outer layer of fragments was weaker than that on the inner layer. The maximum initial velocity of fragments was located near the center of the warhead where the sparse waves intersected, located at around 0.66 times the full length of the warhead.

## 4. Detonation-Driven Deformation and Jet Formation

High pressure on materials could be generated by an explosion. The interaction between explosion products and materials is very quick, and the strain rate could be extremely high. Preformed fragments can deform or even fracture when subjected to contact blasts, which might lead to a reduction of the terminal effect [3,4,5]. To solve this problem, Meng et al. [5] analyzed the effect of surface electroplating on the fragment deformation behavior under contact blasts. The results showed that the pressure amplitude of the uncoated samples instantly dropped to zero after the shock wave passed through the far-exploding surface, which resulted in the formation of a tensile zone. But the pressure amplitude of the coated samples increased, transforming the tensile zone into the compression zone, thereby preventing the fracture of the fragment near the far-exploding surface, which was consistent with the test and simulated results.

In addition to deformation and fracture, materials under extreme high pressure also undergo flow and phase changes. Ma et al. [6] analyzed the formation characteristics of the shaped charge jet (SCJ) from the shaped charge with a trapezoid cross-section. A theoretical model was developed to analyze the collapsing mechanism of the liner driven by the charge with a trapezoid cross-section. The results showed that the influence of the angle of the trapezoidal charge on the axial velocity of the SCJ was not distinct, whereas the variation of the radial velocity of the shaped charge jet was obvious as the change in the angle of the trapezoidal charge. Aiming at the dynamic penetration process of a shaped-charge jet, Wang et al. [7] proposed a mathematical model for the penetration of a jet under dynamic conditions, based on the theory of the virtual origin and the Bernoulli equation taking into account the jet and target intensities. The dynamic penetration process of the jet was divided according to the penetration channel. The dynamic penetration model of the jet based on the unperturbed section and perturbed section was established. Zhang et al. [8] studied the forming process of explosively formed projectiles (EFPs) in air and water. They used Euler governing equations to establish numerical models of EFPs subjected to air and underwater explosions. The fitting formulae of velocity attenuation of EFPs, which form and move in different media, were gained.

## 5. Shock-Induced Chemical Reaction

Metal/fluoropolymer-based reactive materials (RMs) would undergo violent energy release reactions under high-velocity impact and high pressure, and their impact-induced chemical reactions have attracted extensive research. Guo et al. [9] systematically researched the mechanical performances, fracture mechanisms, thermal behavior, energy release behavior, and reaction energy of four types of RMs (26.5% Al/73.5% PTFE; 5.29% Al/80% W/14.71% PTFE; 62% Hf/38% THV; 88% Hf/12% THV) by conducting compressive tests, scanning electron microscope (SEM), differential scanning calorimeter, thermogravimetric (DSC/TG) tests and ballistic experiments. The results show that the THV-based RMs have a unique strain-softening effect, whereas the PTFE-based RMs have a remarkable strain-strengthening effect, which is mainly caused by the different glass transition temperatures. Yuan et al. [10] studied the control of the shock-induced energy release characteristics of PTFE/Al-based energetic material by adding oxides (bismuth trioxide, copper oxide, molybdenum trioxide, and iron trioxide) via experimentation and theoretical analysis. Based on these experimental results, an analytical model was developed, indicating that the apparent activation energy and impact shock pressure dominated the energy release characteristic of PTFE/Al/oxide. This controlling mechanism indicated that oxides enhanced the reaction after shock wave unloading, and the chemical and physical properties of the corresponding thermites also affected the energy release characteristics. These conclusions can guide the design of metal/fluoropolymer-based RMs [2,9,10]. 

Sun et al. [11] investigated the mechanical properties, constitutive behaviors, and failure criteria of aluminum–polytetrafluoroethylene–tungsten (Al–PTFE–W) reactive materials with W content from 20% to 80%. Based on the experimental results and numerical iteration, the J–C constitutive (A, B, n, C, and m) and failure parameters (D1~D5) were well-determined. The research results would be useful for the numerical studies, design, and application of reactive materials. Lu et al. [12] proposed an impact-initiated chemical reaction model to describe the ignition and energy release behavior of Al/PTFE RM. The hotspot formation mechanism of pore collapse was first introduced to describe the decomposition process of PTFE. Material fragmentation and PTFE decomposition were used as ignition criteria. Then, the reaction rate of the decomposition product with aluminum was calculated according to the gas–solid chemical reaction model. Finally, the reaction states of RM calculated by the model are compared and qualitatively consistent with the experimental results. The model provided insight into the thermal–mechanical–chemical responses and references for the numerical simulation of the impact ignition and energy release behavior of RMs.

## 6. Structural Damage and Material Failure Caused by High-Velocity Impact

The deformation and failure of materials under high pressure are related to structural optimization and equipment design [4,8,13,14]. The electronic components inside a main battle tank (MBT) are the key components by which the tank exerts its combat effectiveness. An explicit nonlinear dynamic analysis was performed to study the vibration features and fault mechanism under instantaneous shock load by Li et al. [13]. They obtained the curves of stress–frequency and strain–frequency of the CPU board under different harmonic loads, which were applied to further identify the peak response of the structure. Validation of the finite element model and simulation results are performed by comparing those obtained from the model with experiments. Based on dynamic simulation and experimental analysis, fault patterns of the CPU board were discussed, and some optimization suggestions were proposed.

The space harpoon is a rigid–flexible coupled debris capture method with a simple, reliable structure and high adaptability to the target. Zhao et al. [14] studied the process of impacting and embedding the harpoon into the target plate, the effect of friction at a low-velocity impact, and the criteria for the effective embedding of the harpoon. A simulation model of the dynamics of the harpoon and the target plate considering tangential friction was established, and the reliability of the numerical simulation model was verified by comparing the impact test. The results showed that the frictional effect in the low-velocity impact was more obvious for the kinetic energy consumption of the harpoon itself, and the effective embedding of the harpoon into the anchored target ranges from 50 to ~90 mm, corresponding to a theoretical launch initial velocity between 88.4 and ~92.5 m/s.

This Special Issue includes a small number of discrete case studies on the topic of “Materials under High Pressure”. We hope that this Special Issue will be of interest to the academic community, and the valuable contributions from all the authors are highly appreciated.
